# How does leaf succulence relate to plant drought resistance in woody shrubs?

**DOI:** 10.1093/treephys/tpad066

**Published:** 2023-05-19

**Authors:** Bihan Guo, Stefan K Arndt, Rebecca E Miller, Christopher Szota, Claire Farrell

**Affiliations:** School of Agriculture, Food and Ecosystem Sciences, Faculty of Science, The University of Melbourne, 500 Yarra Boulevard, Richmond, Victoria 3121, Australia; School of Agriculture, Food and Ecosystem Sciences, Faculty of Science, The University of Melbourne, 500 Yarra Boulevard, Richmond, Victoria 3121, Australia; School of Agriculture, Food and Ecosystem Sciences, Faculty of Science, The University of Melbourne, 500 Yarra Boulevard, Richmond, Victoria 3121, Australia; Royal Botanic Gardens Victoria, Birdwood Avenue, Melbourne, Victoria 3004, Australia; School of Agriculture, Food and Ecosystem Sciences, Faculty of Science, The University of Melbourne, 500 Yarra Boulevard, Richmond, Victoria 3121, Australia; School of Agriculture, Food and Ecosystem Sciences, Faculty of Science, The University of Melbourne, 500 Yarra Boulevard, Richmond, Victoria 3121, Australia

**Keywords:** degree of iso-anisohydry, desiccation, plant drought response, plant–water relations, stored water

## Abstract

Succulence describes the amount of water stored in cells or organs, regardless of plant life-form, including woody and herbaceous plants. In dry environments, plants with greater survival often have greater leaf succulence. However, it is unclear how leaf succulence relates to plant drought resistance strategies, including isohydry (closing stomata to maintain leaf water status) and anisohydry (adjusting cell turgor to tolerate low leaf water status), which exist on a continuum that can be quantified by hydroscape area (larger hydroscape area indicates more anisohydric). We evaluated 12 woody species with differing leaf succulence in a glasshouse dry-down experiment to determine relationships among leaf succulence (degree of leaf succulence, leaf succulent quotient and leaf thickness) and plant drought response (hydroscape area, plant water use, turgor loss point and predawn leaf water potential when transpiration ceased). Hydroscape areas ranged from 0.72 (*Carpobrotus modestus* S.T.Blake; crassulacean acid metabolism (CAM) plants) to 7.01 MPa^2^ (*Rhagodia spinescens* R.Br.; C_3_ plants), suggesting that *C. modestus* was more isohydric and *R. spinescens* was more anisohydric. More isohydric species *C. modestus*, *Carpobrotus rossii* (Haw.) Schwantes and *Disphyma crassifolium* (L.) L.Bolus (CAM plants) had greater leaf succulence, lower root allocation, used stored water and ceased transpiration at higher predawn leaf water potential, shortly after reaching their turgor loss point. The remaining nine species that are not CAM plants had larger hydroscape areas and ceased transpiration at lower predawn leaf water potential. Greater leaf succulence was not related to cumulative water loss until transpiration ceased in drying soils. All 12 species had high turgor loss points (−1.32 to −0.59 MPa), but turgor loss point was not related to hydroscape area or leaf succulence. Our data suggest that overall greater leaf succulence was related to isohydry, but this may have been influenced by the fact that these species were also CAM plants.

## Introduction

Many plants in dry environments, like *Agave* and *Opuntia* spp., have succulent tissues that store water and ensure survival in drying soils, and these plants often have thickened leaves and stems adopting the crassulacean acid metabolism (CAM) photosynthesis pathway (Smith et al. 1997, Eggli and Nyffeler 2009, Ramawat 2009). However, as a trait, ‘succulence’ exists in all type of plants and describes the water storage capacity of plant cells or organs including leaves, stems and roots (Von Willert et al. 1992, Eggli and Nyffeler 2009, Ogburn and Edwards 2012). The degree of leaf succulence, which expresses water storage per unit leaf area, and leaf thickness are the most frequently used leaf succulence measures (Delf 1912, Teeri et al. 1981, Griffiths et al. 2008), although Von Willert et al. (1992) suggested that the leaf succulence quotient is a superior measure of leaf succulence as it takes water stored structurally in plant tissues into account. CAM plants with thickened leaves or stems use stored water to maintain transpiration and reproduction, and delay desiccation under drought conditions (Nobel 1976, 1977, 2006). These plants reallocate stored water from parenchyma tissues to maintain transpiration in the first few days of drying events and later reallocate water to inflorescences for reproduction or other tissues to maintain cuticular transpiration (Nobel 1976, 1977, 2006). In arid or coastal environments, leaf succulence occurs as a trait in woody shrubs and subshrubs; however, the role of leaf succulence in improving drought survival is unclear. It has been hypothesized that woody plants with greater leaf succulence have greater drought survival (Blackman et al. 2019). Accordingly, when comparing desiccation time in three woody angiosperms and a conifer (C_3_ plants), the conifer *Pinus radiata* had a longer desiccation time than angiosperms due to greater leaf succulence, early stomatal closure and lower cuticular transpiration (Blackman et al. 2019). In a study comparing drought resistance of different *Eucalyptus* clones (*Corymbia citriodora*, *C. torelliana*, *Eucalyptus cloeziana*, *E. camaldulensis*, *E. grandis* and *E. urophylla*; C_3_ plants), clones with higher values of leaf succulence maintained higher leaf relative water content and less negative leaf water potential under drought conditions (Reis et al. 2021). Also, the root succulence of *Atriplex halimus* (C_4_ shrub) has been shown to assist in coping with salinity (Nedjimi et al. 2006), which can lead to physiological drought (Munns 2002), suggesting that greater leaf succulence in C_4_ woody plants may delay desiccation. These studies indicate that woody plants with greater leaf succulence may close stomata at less negative leaf water potentials and use stored water to maintain leaf water content and delay desiccation.

While succulence may improve drought survival due to greater water storage, it is unclear how succulence as a trait for water storage relates to drought resistance strategies (Sack and Holbrook 2006, Eggli and Nyffeler 2009, Preisler et al. 2021). In perennial plants, there are two main drought response strategies: the isohydric strategy, where plants close their stomata to maintain relatively higher tissue water content (less negative leaf water potential), and the anisohydric strategy, where plants tolerate lower tissue water content (more negative leaf water potential) by adjusting cell turgor (Levitt 1980, Tardieu and Simonneau 1998, Delzon 2015). The degree of isohydry and anisohydry is considered as a continuum, with species that are more isohydric at one end and more anisohydric at the other (Martínez-Vilalta et al. 2014, Meinzer et al. 2016, Johnson et al. 2018). One of the metrics that quantifies the degree of isohydry and anisohydry is the hydroscape area, which incorporates the range of predawn (Ѱ_pd_) and midday leaf water potentials (Ѱ_md_) where stomata control leaf water potential in drying soil (Meinzer et al. 2016, Johnson et al. 2018). Additionally, plants with more negative turgor loss points (Ψ_TLP_) are also considered to be more anisohydric (Bartlett et al. 2012*b*, Meinzer et al. 2016, Blackman 2018, Zhu et al. 2018, Li et al. 2019). Generally, CAM plants with thickened leaves or stems (e.g., *Agave* spp.) are more isohydric as they close stomata, have low cuticular transpiration and metabolic activity and use stored water to maintain water status in drying soils (Nobel 1977, Hanscom and Ting 1978, Levitt 1980, Pimienta-Barrios et al. 2002). Beyond these plants, however, there is little information on how succulence relates to drought resistance strategies in woody plants.

We investigated the drought resistance strategies of 12 woody shrubs and subshrubs with varying degrees of leaf succulence. These 12 species included three subshrubs that are CAM plants with thickened leaves and/or stems. We hypothesized that woody plants with greater leaf succulence (i) will be more isohydric; (ii) will cease transpiration at higher leaf water potentials in drying soils; and (iii) will avoid drought stress by using stored water to maintain water status (predawn and midday leaf water potentials).

## Materials and methods

### Plant selection and plant establishment

We selected 12 woody shrubs and subshrubs from the families Aizoaceae and Amaranthaceae that varied according to their leaf succulence. These included three CAM plants with thickened leaves and/or stems—*Carpobrotus modestus* S.T.Blake, *C. rossii* (Haw.) Schwantes and *Disphyma crassifolium* (L.) L.Bolus; and nine halophyte shrubs (Agriculture Victoria 2022, Royal Botanic Gardens Victoria 2022)—*A. cinerea* Poir., *A. nummularia* Lindl., *A. paludosa* R.Br., *A. semibaccata* R.Br., *Enchylaena tomentosa* R.Br., *Maireana oppositifolia* (F.Muell.) Paul G.Wilson, *Rhagodia candolleana* Moq., *R. spinescens* R.Br. and *Tetragonia implexicoma* (Miq.) Hook.f. (Table 1).

**Table 1:** Description of the 12 selected species, including their family, life form, photosynthetic type and habitat. Species codes are used in the figures.

Family^7^^,^^9^	Species^7^^,^^9^^,^^10^	Species codes	Life form^3^^,^^7^^,^^9^^,^^11^	Photosynthetic type^2^^,^^4^^,^^5^^,^^6^^,^^8^^,^^12^	Habitat^1^^,^^7^
Aizoaceae	*Carpobrotus modestus* S.T.Blake	*Cm*	Evergreen subshrub	CAM/C_3_	Shallow, rocky or deep sandy soils
	*Carpobrotus rossii* (Haw.) Schwantes	*Cr*	Evergreen subshrub	CAM/C_3_	Coastal dunes, banksia woodland and sandy soils
	*Disphyma crassifolium* (L.) L.Bolus	*Dc*	Evergreen subshrub	CAM/C_3_	Coastal and inland, especially in saline soils
	*Tetragonia implexicoma* (Miq.) Hook.f.	*Ti*	Evergreen shrub	C_3_	Coastal dunes, saltmarshes and banksia woodland
Amaranthaceae	*Atriplex cinerea* Poir.	*Ac*	Evergreen shrub	C_4_	Shorelines and dunes, occasionally further inland
	*Atriplex nummularia* Lindl.	*An*	Evergreen shrub	C_4_	Heavy, alkaline soils
	*Atriplex paludosa* R.Br.	*Ap*	Evergreen shrub	C_4_	Saline marshy area, common not only near the coast but also further inland
	*Atriplex semibaccata* R.Br.	*As*	Evergreen shrub	C_4_	Roadsides or other disturbed areas
	*Enchylaena tomentosa* R.Br.	*Et*	Evergreen shrub	C_3_	Slightly saline soils
	*Maireana oppositifolia* (F.Muell.) Paul G.Wilson	*Mo*	Evergreen shrub	C_3_	Coastal and inland areas, including coastal mud flats
	*Rhagodia candolleana* Moq.	*Rc*	Evergreen shrub	C_3_	Coastal or saline areas
	*Rhagodia spinescens* R.Br.	*Rs*	Evergreen shrub	C_3_	Dry habitats

^1^ Agriculture Victoria Resources Online

^2^ Ahmad and Ismail (2002)

^3^ Australian Bureau of Flora, Australian Biological Resources Study (2002)

^4^ De Villiers (1996)

^5^ Farrell et al (2012)

^6^ Prider and Facelli (2004)

^7^ Royal Botanic Gardens Victoria Flora of Victoria

^8^ The University of Sussex Salt-tolerant Plant Data Base

^9^ University of Melbourne Burnley Plant Guide

^10^ Western Australian Herbarium Florabase

^11^ Wilcox D (2015)

^12^ Winter et al (1981)

Plants (except *R. spinescens*) of the same age were obtained as tube stock (plugs) from three commercial nurseries (Westgate Biodiversity: Bili Nursery & Landcare, Goldfields Revegetation and Wildtech Nursery Pty Ltd; Australia) in December 2019. We propagated *R. spinescens* from cuttings in December 2019. We transplanted tube stock plants on 23 June 2020 (winter) into pots (25-cm diameter, 27.5-cm deep; one individual per pot) filled with 6-kg potting mix (parts by volume: 1 part coarse mined sand and 4 parts medium pine bark; additives per m^3^: 4 kg Debco Greenjacket No. 2 slow-release fertilizer (N:P:K 16.5:4.1:9.6), 1.5-kg granular soil wetter SaturAid and 1000-g dolomite lime; air-filled porosity = 9.3 ± 0.3%, bulk density = 0.47 ± 0.007 g cm^−3^, water-holding capacity = 54 ± 0.5%, permanent wilting point = 9.1% of soil water content and electrical conductivity = 1.05 ± 0.02 dS m^−1^; Richards et al. 2017). Plants were grown outside in the Nursery of Burnley Campus, the University of Melbourne (latitude 37°47’S; longitude 144°58’E) and were well watered through a spray irrigation system (06:50, 13:58 and 17:00 h; 1.3 l water per time per pot) for 9 months to ensure that they were established before the dry-down experiment, initial harvest and pressure–volume (PV) curve measures.

### Dry-down experiment

Five replicate pots per species were moved to an enclosed rainout shelter in Burnley Campus for the dry-down experiment (9 March 2021) and arranged in a complete randomized block design. Initially, all pots were watered three times a day with an automatic irrigation system (07:00, 13:00 and 17:00 h; 1.3 l water per pot for each watering). In the evening (19:00 h) before the dry-down commenced, all pots were watered by hand to field capacity. We stopped watering on 17 March 2021 and started the dry-down experiment, which finished on 16 August 2021. All pots were not covered, and water loss included evaporation and transpiration. Over this period, the daytime (07:00–19:00 h) average temperature was 19.5 ± 0.03 °C, average humidity was 90.8 ± 0.0006%; the night-time (19:00–07:00 h) average temperature was 13.3 ± 0.03 °C and average humidity was 95.4 ± 0.0006%. The vapor pressure deficit (VPD; KPa) was calculated as (Marsh et al. 2022) 


(1) \begin{equation*} \frac{100-\mathrm{relative}\ \mathrm{humidity}}{100}\times \frac{610.7\times{10}^{\frac{7.5\times \mathrm{temperature}}{237.3+\mathrm{temperature}}}}{1000}. \end{equation*}

Cumulative VPD (KPa h) was determined as the addition of VPD multiplied by time (hours).

### Measurements

#### Plant water use and leaf water potential measurements

During the dry-down, pots were weighed at predawn (05:30 h) every weekday from 18 March to 2 April 2021; every second day from 3 April to 12 April 2021; and once a week from 13 April to 16 August 2021. Daily evapotranspiration (daily ET; kg day^−1^) was determined as (Somerville et al. 2019)


(2) \begin{equation*} \frac{\mathrm{pot}\ \mathrm{weight}\ \mathrm{of}\ \mathrm{the}\ \mathrm{previous}\ \mathrm{day}-\mathrm{pot}\ \mathrm{weight}\ \mathrm{of}\ \mathrm{the}\ \mathrm{day}}{\mathrm{number}\ \mathrm{of}\ \mathrm{days}\ \mathrm{between}\ \mathrm{days}}. \end{equation*}

Predawn leaf water potential (Ψ_pd_) was measured on terminal shoots sampled from pots weighed at predawn (05:30 h) every weekday from 18 March to 2 April 2021; every second day from 3 April to 12 April 2021; and once a week from 13 April to 16 August. Midday leaf water potential (Ψ_md_) was measured on terminal shoots sampled from each pot at 13:00 h on the same days. Both Ψ_pd_ and Ψ_md_ were measured using a Scholander-type pressure chamber (Soilmoisture Equipment Corp., Santa Barbara, CA, USA).

#### Leaf water potential at the turgor loss point (Ψ_TLP_)

We determined Ψ_TLP_ (MPa) from PV curves measured on well-watered plants from February to August 2021. One terminal shoot from each well-watered replicate (six replicates per species) was collected in the early morning (06:30–07:00 h) and recut under distilled water. We then placed the shoots in 50-ml centrifuge tubes with distilled water to rehydrate the shoots for 2–3 h or until leaf water potentials were above −0.1 MPa. Rehydrated shoots were weighed before measuring leaf water potential with the Scholander-type pressure chamber. We repeated the weighing and leaf water potential measurements as samples dried in the laboratory. After finishing the measurements, samples were oven-dried at 80 °C until reaching a constant weight to determine the dry mass. Ψ_TLP_ was determined using the PV curve fitting Excel v5.6 developed by K.P.Tu and J.B.Fisher (http://landflux.org/Tools.php) based on Schulte and Hinckley (1985).

#### Plant morphological traits (leaf succulence, root mass fraction and leaf mass per area)

Plant morphological traits were measured on well-watered plants with five pots per species when dry-down started. Soil was removed from roots, and shoots were separated into leaves and stems to determine fresh and dry weight of leaves, stems and roots. Leaf areas were measured using a LI3100 area meter (Licor, Lincoln, NE, USA). Samples were placed in an 80 °C oven for a week until reaching a constant weight, after which dry weights were measured. Leaf ash weight was determined by burning oven-dried leaves in a 500 °C muffle furnace for 2 h, and this process was repeated until they reached constant weight.

We determined three measures of leaf succulence: leaf thickness, leaf succulence quotient and degree of leaf succulence. Leaf thickness (mm) was measured on the midpoint of freshly picked leaves using electronic Vernier calipers. Leaf succulence quotient (g H_2_O cm^−2^ leaf area g^−1^ organic matter cm^−2^ leaf area) was determined as (Von Willert et al. 1992)


(3) \begin{equation*} \frac{\mathrm{fresh}\ \mathrm{weight}- \mathrm{dry}\ \mathrm{weight}}{\mathrm{leaf}\ \mathrm{area}}\div \frac{\mathrm{dry}\ \mathrm{weight}- \mathrm{ash}\ \mathrm{weight}}{\mathrm{leaf}\ \mathrm{area}}. \end{equation*}

Degree of leaf succulence (g H_2_O cm^−2^ leaf area) was calculated as (Delf 1912)


(4) \begin{equation*} \frac{\mathrm{leaf}\ \mathrm{fresh}\ \mathrm{weight}- \mathrm{leaf}\ \mathrm{dry}\ \mathrm{weight}}{\mathrm{leaf}\ \mathrm{area}\ }. \end{equation*}

Root mass fraction (RMF; g root dry mass g^−1^ plant dry mass) was determined as (Pérez-Harguindeguy et al. 2013)


(5) \begin{equation*} \frac{\mathrm{root}\ \mathrm{dry}\ \mathrm{mass}}{\mathrm{total}\ \mathrm{plant}\ \mathrm{dry}\ \mathrm{mass}}. \end{equation*}

Leaf mass per area (LMA; g leaf dry mass m^−2^ leaf area) was determined as


(6) \begin{equation*} \frac{\mathrm{leaf}\ \mathrm{dry}\ \mathrm{mass}}{\mathrm{leaf}\ \mathrm{area}}. \end{equation*}

### Data analysis

All data analyses were processed using R (R studio team 2016). Normality was tested using histograms and Q-Q plots.

#### Cumulative VPD where evapotranspiration ceased (*E*_0_)

We plotted daily ET (*y*-axis) against cumulative VPD (*x*-axis). Gamma distribution with the inverse link was selected for generalized linear regression models (GLMs) based on the comparison of AIC scores (Model 1). The point where evapotranspiration ceased (*E*_0_) was where the curve crossed the axis of symmetry (*y* = 1/*x* is symmetric to *y* = *x*). Daily ET at *E*_0_ (kg d^−1^) was determined as the *y* value of *E*_0_, and cumulative VPD at *E*_0_ (KPa h) was determined as the *x* value of *E*_0_.

#### Cumulative VPD when Ψ_TLP_ was reached

To determine the time when plants reached their Ψ_TLP_, we first plotted daily ET (*y*-axis) against their corresponding Ψ_pd_ (*x*-axis; Model 2). Gamma distribution was used for GLMs. We compared the AIC scores for models using different link functions and selected the exponential function. We then used the Ψ_TLP_ (MPa; *x* value) and the exponential model (Model 2) to predict the daily ET at Ψ_TLP_ (kg day^−1^; *y* value). We then used daily ET at Ψ_TLP_ (kg day^−1^) as y value for Model 1, the GLM model we built for daily ET (kg day^−1^) and cumulative VPD (KPa h), to determine cumulative VPD when Ψ_TLP_ was reached (KPa h).

#### Cumulative water loss until plants reached *E*_0_ (cumulative ET at *E*_0_)

We fitted an inverse function to the relationship between pot weight (kg; *y*-axis) for each predawn measurement and cumulative VPD (KPa h; *x*-axis) using the same approach described above (Model 3). The cumulative VPD at *E*_0_ (KPa h; *x* value) and the fitted Model 3 were used to determine the pot weight at *E*_0_ (kg; *y* value). The cumulative ET at *E*_0_ (kg) was calculated as the initial pot weight (kg) − the pot weight at *E*_0_ (kg). We then adjusted cumulative water loss before plants ceased evapotranspiration by initial shoot dry mass (cumulative ET at *E*_0_/shoot dry mass; kg H_2_O kg^−1^ shoot dry mass) and leaf area (cumulative ET at *E*_0_/leaf area; kg H_2_O m^−2^ leaf area), as plants did not lose many leaves when plants ceased evapotranspiration.

#### Predawn leaf water potential at *E*_0_ (Ψ_pd_ at *E*_0_) and the difference in predawn leaf water potential between *E*_0_ and Ψ_TLP_ (ΔΨ_pd_ between *E*_0_ and Ψ_TLP_)

The predawn leaf water potential at *E*_0_ (Ψ_pd_ at *E*_0_; MPa; *x* value) was predicted by the daily ET at *E*_0_ (kg day^−1^; *y* value) using Model 2; the GLM exponential model we built using the relationship between daily ET (kg day^−1^; *y*-axis) and Ψ_pd_ (MPa; *x*-axis). The predawn leaf water potential difference between *E*_0_ and Ψ_TLP_ (MPa) was then calculated as Ψ_pd_ at *E*_0_ (MPa) − Ψ_TLP_ (MPa).

#### Degree of isohydry and anisohydry (hydroscape areas)

We used hydroscape area (MPa^2^) to quantify isohydric or anisohydric strategies (more isohydric species have a smaller hydroscape area; Martínez-Vilalta et al. 2014, Meinzer et al. 2016, Johnson et al. 2018). As per Johnson et al. (2018), a convex hull was used to capture the hydroscape area. However, only paired measurements of Ψ_pd_ and Ψ_md_ captured while plants were actively transpiring (Ψ_pd_ ≥ Ψ_pd_ at *E*_0_) were used to quantify the hydroscape area. This approach was similar to one approach taken by Meinzer et al. (2016), where data were only included while stomata were actively regulating water potential. We suggest this may avoid overestimating the hydroscape area as it removes measurements collected when plants are effectively desiccated.

#### Plant morphological traits, functional groups and relationships among traits

We conducted principal component analysis (PCA) to investigate variation in multiple traits among the 12 species based on morphological traits including LMA, RMF, degree of leaf succulence, leaf thickness and leaf succulence quotient, and on traits associated with drought resistance strategies including hydroscape area, Ψ_pd_ at *E*_0_, cumulative VPD at *E*_0_, cumulative ET at *E*_0_ and Ψ_TLP_. The 12 species were then grouped using cluster analyses (hierarchical and *k*-means), and the optimal number of clusters was selected using the ‘elbow’ method (Thorndike 1953). Individual relationships among traits or subgroups were determined by Pearson correlations and PCA axes.

## Results

### Plant water use and water status

#### Cumulative VPD where evapotranspiration ceased (*E*_0_)

**Figure 1:**
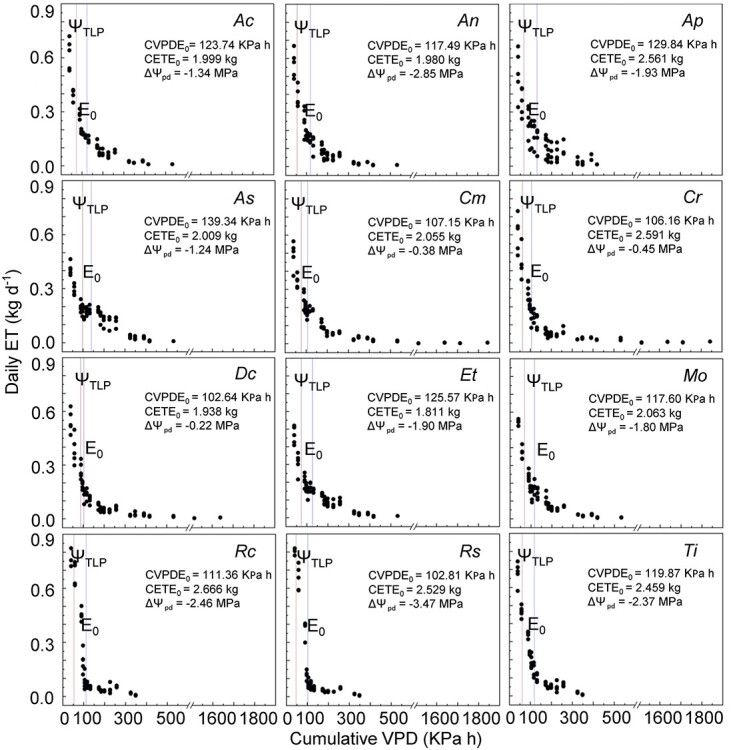
Change in daily water use (daily ET; kg day^−1^) over time (represented by cumulative VPD; abbreviation: CVPDE_0_; KPa h). The blue lines indicate the cumulative VPD until ET ceased (cumulative VPD at E_0_), and the red lines indicate the cumulative VPD until the turgor loss point (Ψ_TLP_). Species codes (*italic*; in the top right corner of each graph) are in Table 1. Cumulative VPD at *E*_0_ (abbreviation: CVPDE_0_; KPa h), cumulative water loss until *E*_0_ (cumulative ET at *E*_0_; abbreviation: CETE_0_; kg), and the difference in leaf water potential between *E*_0_ and Ψ_TLP_ (ΔΨ_pd_ between *E*_0_ and Ψ_TLP_; abbreviation: ΔΨ_pd_; MPa) are reported below the species codes. All species except *Cm, Cr* and *Dc* died before cumulative VPD reached 600 KPa h, and as the slopes of *Cm*, *Cr* and *Dc* after 600 KPa h did not change, an axis break was used between 600 and 1500 KPa h.

The 12 species varied in the time it took to cease evapotranspiration (cumulative VPD at *E*_0_; Figure 1 and see Table S1 available as Supplementary data at *Tree Physiology* Online). *Disphyma crassifolium* ceased evapotranspiration first (cumulative VPD at *E*_0_ = 102.6 KPa h) and *A. semibaccata* and *A. paludosa* took the longest time to cease evapotranspiration (cumulative VPD at *E*_0_ for *A. semibaccata* = 139.34 KPa h; for *A. paludosa* = 129.8 KPa h).

#### Cumulative water loss until evapotranspiration ceased

The amount of water loss before plants reached *E*_0_ (cumulative ET at *E*_0_) also varied among the 12 species (Figure 1 and see Table S1 available as Supplementary data at *Tree Physiology* Online). The highest water spenders were *R. candolleana* (cumulative ET at *E*_0_ = 2.7 kg), *C. rossii* (2.6 kg), *A. paludosa* (2.6 kg), *R. spinescens* (2.5 kg) and *T. implexicoma* (2.5 kg). The lowest water spenders included *E. tomentosa* (cumulative ET at *E*_0_ = 1.8 kg), *D. crassifolium* (1.9 kg), *A. nummularia* (2.0 kg), *A. cinerea* (2.0 kg), *A. semibaccata* (2.0 kg), *C. modestus* (2.1 kg) and *M. oppositifolia* (2.1 kg). However, the cumulative water loss per shoot dry mass for *A. semibaccata* was the highest (cumulative ET at *E*_0_/shoot dry mass = 42.33 kg H_2_O kg^−1^ shoot dry mass) and that for *A. nummularia* was the lowest (19.91 kg H_2_O kg^−1^ shoot dry mass; see Table S1 available as Supplementary data at *Tree Physiology* Online). The ranks changed when adjusting the cumulative water loss by leaf area, where *C. modestus* was the highest water spender (cumulative ET at *E*_0_/leaf area = 139.05 kg H_2_O m^−2^ leaf area) and *M. oppositifolia* was the lowest water spender (30.99 kg H_2_O m^−2^ leaf area).

#### Cumulative VPD when Ψ_TLP_ was reached

All plants were still transpiring at their turgor loss point (Ψ_TLP_), and some plants closed stomata at much lower Ψ_pd_ compared with Ψ_TLP_ (−ΔΨ_pd_ between *E*_0_ and Ψ_TLP_; Figure 1). Some of the highest water spenders also had the greatest changes in predawn leaf water potential between *E*_0_ and Ψ_TLP_, including *R. candolleana* (−ΔΨ_pd_ between *E*_0_ and Ψ_TLP_ = 2.46 MPa), *A. paludosa* (1.93 MPa), *R. spinescens* (3.47 MPa) and *T. implexicoma* (2.37 MPa). Although *C. rossii* was also one of the greatest water spenders, it ceased evapotranspiration very close to its Ψ_TLP_ (−ΔΨ_pd_ between E_0_ and Ψ_TLP_ = 0.45 MPa). The lowest water spenders differed in their maintenance of water status. Both *C. modestus* and *D. crassifolium* ceased transpiration at Ψ_pd_ close to their Ψ_TLP_ (−ΔΨ_pd_ between E_0_ and Ψ_TLP_ for *C. modestus* = 0.38 MPa and *D. crassifolium* = 0.22 MPa), whereas *A. nummularia* and *E. tomentosa* had the largest changes in water status between E_0_ and Ψ_TLP_ (−ΔΨ_pd_ between E_0_ and Ψ_TLP_ for *A. nummularia* = 2.85 MPa and *E. tomentosa* = 1.90 MPa).

### Degree of isohydry and anisohydry

**Figure 2:**
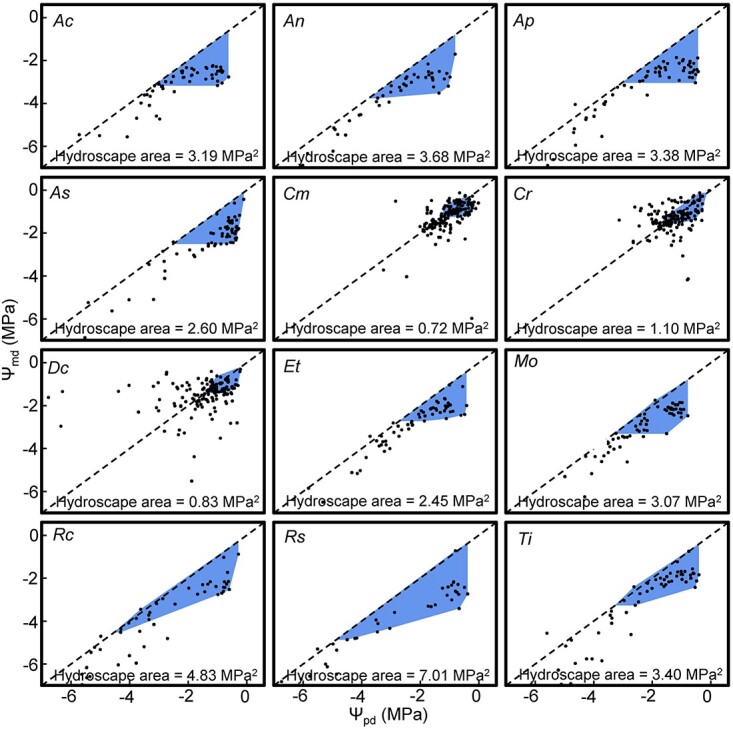
Midday leaf water potential (Ψ_md_; MPa) in relation to predawn leaf water potential (Ψ_pd_; MPa). Blue areas indicate the hydroscape area (MPa^2^) above the value of Ψ_pd_ where evapotranspiration has effectively ceased (*E*_0_). The hydroscape area for each species is written at the bottom of each graph. The larger the blue area, the larger the hydroscape area. Species codes are shown in Table 1.

**Figure 3:**
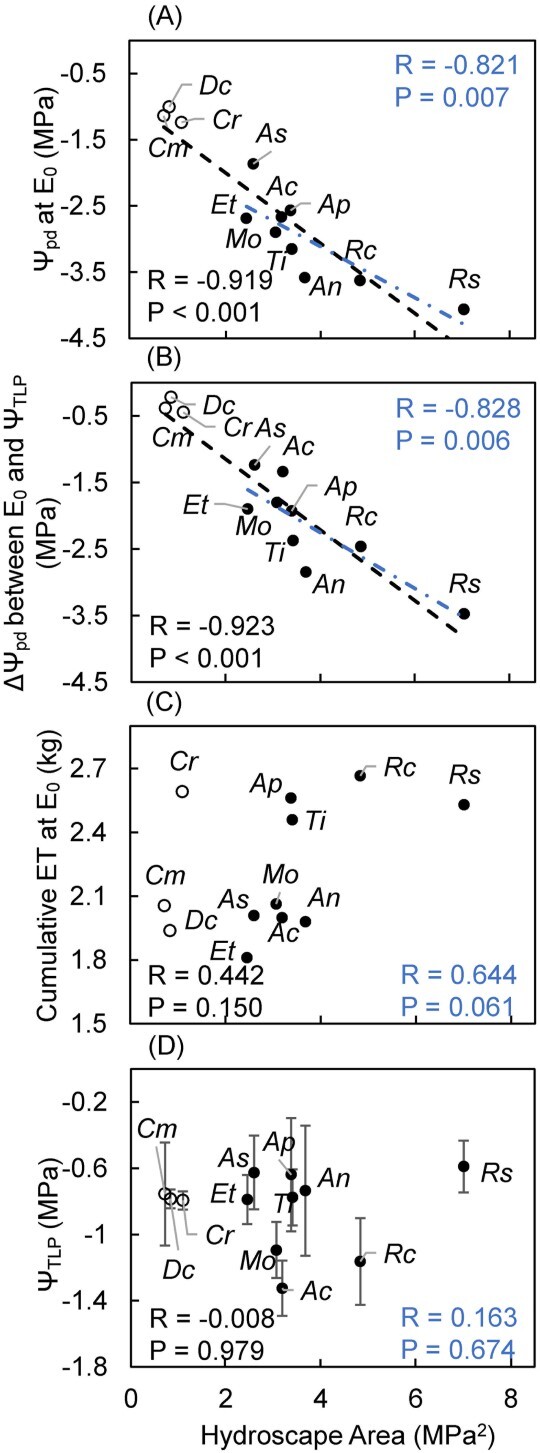
Relationships between hydroscape area (MPa^2^) and (A) predawn leaf water potential when evapotranspiration ceased (Ψ_pd_ at E_0_; MPa), (B) the difference in predawn leaf water potential between E_0_ and Ψ_TLP_ (ΔΨ_pd_ between E_0_ and Ψ_TLP_; MPa), (C) cumulative water loss until evapotranspiration ceased (cumulative ET at *E*_0_; kg) and (D) Ψ_TLP_ (MPa). Species codes are shown in Table 1. Open and filled circles represent two different drought resistance strategy groups; open circles represent species that are more isohydric and filled circles represent species that are more anisohydric. Black dash trendlines show significant relationships among all species, and blue long dash dot trendlines show significant relationships among species except *Cm*, *Cr* and *Dc. R-* and *P*-values for all species (Pearson correlation) are shown in black text at the bottom of each graph. *R-* and *P*-values for species except *Cm*, *Cr* and *Dc* (Pearson correlation) are shown in blue text in each graph.

**Figure 4:**
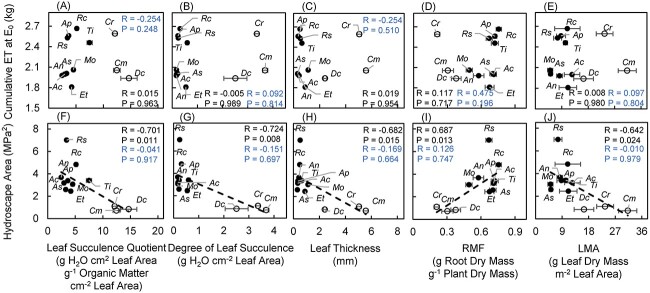
Relationships between cumulative water loss until evapotranspiration ceased (cumulative ET at *E*_0_; kg) and (A) the leaf succulence quotient (g H_2_O cm^2^ leaf area g^−1^ organic matter cm^−2^ leaf area), (B) degree of leaf succulence (g H_2_O cm^−2^ leaf area), (C) leaf thickness (mm), (D) root mass fraction (RMF; root dry mass g^−1^ plant dry mass) and (E) leaf mass per area (LMA; g leaf dry mass m^−2^ leaf area); and relationships between hydroscape area (MPa^2^) and (F) the leaf succulence quotient (g H_2_O cm^2^ leaf area g^−1^ organic matter cm^−2^ leaf area), (G) degree of leaf succulence (g H_2_O cm^−2^ leaf area), (H) leaf thickness (mm), (I) RMF (root dry mass g^−1^ plant dry mass) and (J) leaf mass per area (LMA; g leaf dry mass m^−2^ leaf area). Species codes are shown in Table 1. Open and filled circles represent two different drought resistance strategy groups; open circles represent species that are more isohydric, and filled circles represent species that are more anisohydric. Black dash trendlines show significant relationships among all species*. R-* and *P*-values for all species (Pearson correlation) are shown in black text, and *R-* and *P-*values for species except *Cm*, *Cr* and *Dc* are shown in blue text.

Hydroscape areas ranged between 0.72 and 7.01 MPa^2^. *Carpobrotus modestus* (0.72 MPa^2^), *D. crassifolium* (0.83 MPa^2^) and *C. rossii* (1.10 MPa^2^) had the smallest hydroscape areas (Figure 2 and see Table S1 available as Supplementary data at *Tree Physiology* Online), which indicated that they were more isohydric. *Rhagodia spinescens* (7.01 MPa^2^) and *R. candolleana* (4.83 MPa^2^) had the largest hydroscape areas and were more anisohydric. Based on their hydroscape areas, we divided the 12 species into two groups; a group including *C. modestus*, *C. rossii* and *D. crassifolium* (more isohydric; shown as open dots in Figures 3 and 4) and a group with the remaining nine species (more anisohydric; shown as filled-in dots in Figures 3 and 4).

### Relationships between degree of isohydry and anisohydry (hydroscape area) and plant water use (cumulative water loss until evapotranspiration ceased) or other drought resistance metrics (Ψ_TLP_, Ψ_pd_ at *E*_0_, ΔΨ_pd_ between *E*_0_ and Ψ_TLP_)

There was a strongly significant relationship between hydroscape area and both Ψ_pd_ at E_0_ and ΔΨ_pd_ between E_0_ and Ψ_TLP_ among all tested species as well as when the three CAM plants were excluded (without *C. modestus*, *C. rossii* and *D. crassifolium*; Figure 3A and B), with plants with greater hydroscape areas (more anisohydric) ceasing evapotranspiration at more negative leaf water potentials (Ψ_pd_ at E_0_) and at much lower leaf water potentials than their Ψ_TLP_ (ΔΨ_pd_ between *E*_0_ and Ψ_TLP_). However, there were no significant relationships between hydroscape area and either water use (cumulative ET at *E*_0_) or turgor loss point (Ψ_TLP_) (Figure 3C and D and see Table S2 available as Supplementary data at *Tree Physiology* Online).

### Relationships between morphological traits, water use and hydroscape areas

All measures of succulence, the root mass fraction (RMF) and leaf mass per area (LMA) were strongly correlated with hydroscape area among the 12 species but not water use (cumulative ET at *E*_0_; Figure 4). The three leaf succulence measures (leaf succulence quotient, degree of leaf succulence and leaf thickness) were negatively correlated with hydroscape area, i.e., plants with a greater degree of succulence had smaller hydroscape areas (more isohydric). Conversely, greater RMF and smaller LMA were correlated with greater hydroscape area among the 12 species, meaning that plants with a greater hydroscape area allocated more biomass to roots. However, none of relationships was significant when the three CAM plants (*C. modestus*, *C. rossii* and *D. crassifolium*) were removed from the analysis.

### Variation in morphological and physiological traits among the 12 species

**Figure 5:**
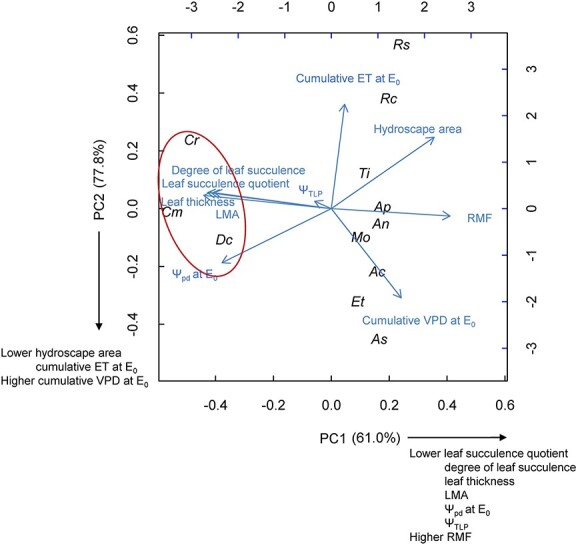
PCA of morphological and physiological traits including leaf succulence quotient (g H_2_O cm^−2^ leaf area g^−1^ organic matter cm^−2^ leaf area), degree of leaf succulence (g H_2_O cm^−2^ leaf area), leaf thickness (mm), leaf mass per area (LMA; g leaf dry mass m^−2^ leaf area), root mass fraction (RMF; g root dry mass g ^−1^ plant dry mass), hydroscape area (MPa^2^), predawn leaf water potential when evapotranspiration ceased (Ψ_pd_ at E_0_; MPa), Ψ_TLP_ (MPa), cumulative VPD where evapotranspiration ceased (cumulative VPD at E_0_; KPa h) and cumulative water loss until evapotranspiration ceased (cumulative ET at E_0_; kg), showing two functional groups (in and out of red ellipse) determined using cluster analysis. Percent variation explained by each axis is shown. Species codes are shown in Table 1.

To explore variation in morphological and physiological traits among the 12 species, we conducted PCA and cluster analysis. The first two principal components explained 77.8% of the variation among species (Figure 5). The first axis (PC1) explained 61.0% of the variation and was negatively correlated with the leaf succulence quotient (*R* = −0.38), degree of leaf succulence (*R* = −0.40), leaf thickness (*R* = −0.39), LMA (*R* = −0.37), Ψ_pd_ at *E*_0_ (*R* = −0.34) and Ψ_TLP_ (*R* = −0.05), and positively correlated with RMF (*R* = 0.37). Therefore, species with smaller PC1 values (*C. modestus*, *C. rossii* and *D. crassifolium*) had a greater leaf succulence quotient, degree of leaf succulence, leaf thickness, LMA, Ψ_pd_ at *E*_0_ and Ψ_TLP_, and smaller RMF. The second axis (PC2) explained 16.8% of the variation and was positively correlated with hydroscape area (*R* = 0.43) and cumulative ET at *E*_0_ (*R* = 0.62) and negatively correlated with cumulative VPD at *E*_0_ (*R* = −0.53). Thus, species with larger PC2 values (e.g., *A. semibaccata*) had greater cumulative VPD at *E*_0_ and smaller hydroscape area and cumulative ET at *E*_0_. Succulence traits (leaf thickness, leaf succulence quotient and degree of succulence) and LMA were highly correlated, and RMF was negatively related to succulence measures and LMA (Figure 5 and see Table S2 available as Supplementary data at *Tree Physiology* Online). Hydroscape area was negatively correlated with Ψ_pd_ at *E*_0_ (Figure 5 and see Table S2 available as Supplementary data at *Tree Physiology* Online). Cluster analysis revealed two groups, with the three CAM plants *C. modestus*, *C. rossii* and *D. crassifolium* grouped together and the other nine species in the second group.

## Discussion

### Drought response strategies of the 12 woody plant species

We hypothesized that woody plants with greater leaf succulence (i) will be more isohydric; (ii) will cease transpiration at higher leaf water potentials in drying soils; and (iii) will avoid drought stress by using stored water to maintain water status (predawn and midday leaf water potentials). We were able to distinguish two groups within the 12 species, based on degree of isohydry and anisohydry: (i) more isohydric species that all were CAM plants: *C. modestus*, *C. rossii* and *D. crassifolium*; and (ii) more anisohydric species: *A. cinerea*, *A. nummularia*, *A. paludosa*, *A. semibaccata*, *E. tomentosa*, *M. oppositifolia*, *R. candolleana* and *R. spinescens* and *T. implexicoma*. The hydroscape areas of the three CAM plants (0.72–1.10 MPa^2^) were the smallest among the 12 species; these plants were more isohydric and ceased transpiration at higher leaf water potentials. This is consistent with our hypothesis and other studies that have shown that CAM plants with thickened leaves or stems are more isohydric (Nobel 1977, Hanscom and Ting 1978, Levitt 1980, Pimienta-Barrios et al. 2002). Isohydry and anisohydry can be considered as a continuum, and while the three CAM plants were the most isohydric, there was a relationship between hydroscape area and leaf succulence for all 12 species. For example, *A. semibaccata* (2.60 MPa^2^)*,* which was a C_4_ plant, was also relatively isohydric compared with other species (2.60 MPa^2^) and closed its stomata at a relatively high predawn leaf water potential (−1.86 MPa). Other plants in the Amaranthaceae that were more isohydric included *E. tomentosa* (2.45 MPa^2^; C_3_ plant), *M. oppositifolia* (3.07 MPa^2^; C_3_ plant) and *A. cinerea* (3.19 MPa^2^; C_4_ plant), whereas *R. candolleana* (C_3_ plant) and *R. spinescens* (C_3_ plant) were the most anisohydric species in our study with large hydroscape areas (*R. candolleana*, 4.83 MPa^2^; *R. spinescens*, 7.01 MPa^2^) and ceased evapotranspiration at the most negative Ψ_pd_ (Ψ_pd_ at *E*_0_; *R. candolleana*, −3.63 MPa; *R. spinescens*, −4.06 MPa). The CAM plants with thickened leaves or stems in our study were strongly isohydric, ceased transpiration at higher leaf water potentials and maintained higher water status during drought which likely confers higher drought resistance.

However, ceasing transpiration to maintain a higher leaf water potential may not necessarily improve the ability of species to survive a drought cycle. The volume of water transpired per unit decrease in leaf water potential differs among species (Goldstein et al. 1984, Meinzer et al. 2003), and the amount of water used before stomatal closure can affect drought survival (McDowell et al. 2008, Caine et al. 2019). However, in our study, cumulative water loss before evapotranspiration ceased (soil water and internal stored water; cumulative ET at *E*_0_) was not significantly related to drought resistance. Since anisohydric species maintain transpiration until lower Ψ, it is reasonable to expect greater water use (Levitt 1980, Tardieu and Simonneau 1998, Delzon 2015, Meinzer et al. 2016); however, the most isohydric species *C. rossii* was one of the largest water spenders and the other CAM species *C. modestus* and *D. crassifolium* also had higher cumulative water loss (either adjusted or not) than most other plants. This is consistent with Chu and Farrell (2022) who showed no difference in cumulative water loss before evapotranspiration ceased in woody plants with both anisohydric (*Teucrium fruticans*, *Jacobaea maritima* and *Rosmarinus officinalis*) and isohydric behavior (*Olearia axillaris*). Therefore, we cannot assume that isohydric species are more conservative in their water use, or vice versa, and therefore should not assume drought resistance based on water use alone.

The turgor loss point has previously been used as a proxy for the degree of iso-anisohydry and therefore an indicator of drought resistance (Bartlett et al. 2016, Meinzer et al. 2016, Li et al. 2019). In our study, all 12 species had very high turgor loss points (Ψ_TLP_; −1.32 to −0.59 MPa), indicating low tolerance of tissue dehydration. All species experienced Ψ_pd_ much lower than Ψ_TLP_, and most species also ceased transpiration at much lower Ψ_pd_ than their Ψ_TLP_. We measured Ψ_TLP_ on well-watered plants, rather than droughted plants, and therefore the measured Ψ_TLP_ on well-watered plants may be higher due to the accumulation of cell solutes or osmotic adjustment (Farrell et al. 2017, Marechaux et al. 2017). Further, we had a relatively narrow range of Ψ_TLP_ values compared with other studies (Bartlett et al. 2016, Meinzer et al. 2016, Li et al. 2019). However, the difference is generally small and insignificant (Farrell et al. 2017, Marechaux et al. 2017). Thus, all these plants had a relatively high Ψ_TLP_, and even if they had adjusted Ψ_TLP_ in response to drought, it will be likely that they still experienced Ψ_pd_ lower than Ψ_TLP_. Species that were more anisohydric (e.g., *R. candolleana* and *R. spinescens*) had larger differences in leaf water potential from Ψ_TLP_ to when evapotranspiration ceased compared with more isohydric species (e.g., *C. modestus*, *C. rossii* and *D. crassifolium*) in our study. There was no relationship between Ψ_TLP_ and predawn leaf water potential when transpiration ceased (Ψ_pd_ at *E*_0_) or hydroscape area (degree of iso-anisohydry). In contrast, the relationship between Ψ_TLP_ and hydroscape area was significant in eight woody species (Meinzer et al. 2016). The lack of a significant relationship in our experiment might be caused by the different measurement method of Ψ_TLP_, as Meinzer et al. (2016) did not rehydrate shoots when determining Ψ_TLP_, but in our experiment, we rehydrated shoots. For anisohydric plants, Ψ_TLP_ measured on rehydrated shoots are often higher than non-rehydrated shoots, while for isohydric species, Ψ_TLP_ measured on rehydrated shoots and on non-rehydrated shoots are often similar (Meinzer et al. 2014). Other studies using the rehydration method have also shown no relationship between Ψ_TLP_ and degree of iso-anisohydry represented by hydroscape area (in 13 tree species evaluated in a dry-down large pot experiment; Thom et al. 2021), by the difference between Ψ_pd_ under well-watered and drought conditions (for 17 Australian native species; Farrell et al. 2017) or by full stomatal closure (in eight tropical dry forest trees; Brodribb et al. 2003). Differences in relationships between Ψ_TLP_ and other measures of drought response strategy may be observed because some species close stomata in response to xylem cavitation and others in response to the change in leaf water potential as mesophyll cell turgor declines (Brodribb et al. 2003). Leaf succulence may also contribute, with high water storage capacitance buffering changes in leaf water potential (Bartlett et al. 2012*a*, 2016, Farrell et al. 2017). Therefore, we suggest that Ψ_TLP_ cannot be used to determine the degree of iso-anisohydry in plants with succulent tissue.

### Relationships between drought resistance and leaf succulence

The three CAM species (*C. modestus*, *C. rossii* and *D. crassifolium*) with the greatest leaf succulence were the most isohydric (smaller hydroscape area) and ceased transpiration at less negative leaf water potentials (Ѱ_pd_ at *E*_0_) that were closer to their Ψ_TLP_ (smaller ΔΨ_pd_ between *E*_0_ and Ψ_TLP_). This is consistent with our hypotheses, as greater leaf succulence means that there is a larger pool of internally stored water in leaves (Eggli and Nyffeler 2009, Ogburn and Edwards 2012) that can be used to maintain water status (Nobel 1976, 2006, Jordan and Nobel 1981). These CAM plants with thickened leaves or stems also had lower root allocation, indicating a trade-off between leaf succulence measures and root allocation. Plants with greater leaf succulence can redirect stored water to maintain less negative root water potentials and keep roots alive; lower root allocation reduces the amount of water needed for protecting roots, and smaller root systems can respond quickly to future rainfall event (Schmidt and Kaiser 1987, Von Willert et al. 1992, North and Nobel 1998, Larcher 2003). For example, *Agave deserti*, the desert CAM plant with thickened leaves, utilized shoot-stored water to maintain root water potentials higher than −2.5 MPa for more than 180 days of drought when soil water potential (determined gravimetrically using a moisture release curve) dropped to ~−35 MPa (North and Nobel 1998). Plants with greater leaf succulence may have also maintained higher ΔΨ_pd_ by refilling tissues while soil water was still available (Von Willert et al. 1992). Therefore, plants with greater leaf succulence may refill their stored water when soil water is available, rely on the leaf succulence when soil water is not available and wait for another rainfall to quickly refill the used stored water through shallow roots.

While greater leaf succulence was associated with greater isohydry in the 12 woody plant species, this relationship did not hold when we removed the three CAM plants (*C. modestus*, *C. rossii* and *D. crassifolium*). This suggests that for the nine other woody species, greater leaf succulence is not associated with isohydric strategies, although these nine species were far less succulent and had more anisohydric behavior with larger hydroscape areas and ceased transpiration at more negative Ψ_pd_ compared with the three CAM plants with thickened leaves and/or stems. These nine species may use their succulent tissues to accumulate sugars for osmotic adjustment to maintain the stomata open at a low Ψ during drought (Levitt 1980, Živčák et al. 2009). The change in the relationship between isohydry and leaf succulence before and after removing CAM plants may also reflect differences in photosynthetic types, as CAM plants can keep their stomata closed during the daytime and undertake CAM photosynthesis at night (Winter and Smith 2012) or use the C_3_ pathway and have gas exchange during the daytime (Orsenigo et al. 1996). This could lead to the differences in transpiration and leaf water potential (Nobel and Jordan 1983, Orsenigo et al. 1996) and therefore hydroscape areas (Nobel and Jordan 1983, Orsenigo et al. 1996). This makes it difficult to accurately determine the hydroscape area of CAM plants, and should be investigated in future studies.

### Relationships among the three leaf succulence measures

The three measures of leaf succulence in our experiment including the degree of leaf succulence (g H_2_O cm^−2^ leaf area), leaf succulence quotient (g H_2_O cm^2^ leaf area g^−1^ organic matter cm^−2^ leaf area) and leaf thickness (mm) were highly correlated to each other. We expected that there would be differences in succulence measures as the leaf succulence quotient describes the stored water per unit organic matter, while the degree of leaf succulence describes the stored water per unit leaf area (Delf 1912, Von Willert et al. 1992). Studies have shown that plants with a larger leaf succulence quotient do not necessarily also have a higher degree of leaf succulence (Von Willert et al. 1990, Jones 2011, Naidoo 2018) due to differences in leaf structure (reflected by ash content) among species (Von Willert et al. 1990). However, in our study, the degree of sclerophylly corrected by ash weight was low, with small variation among species (from 0.04 to 0.30 g organic matter cm^−2^ leaf area; see Table S1 available as Supplementary data at *Tree Physiology* Online) and therefore, the leaf succulence quotient significantly correlated with degree of leaf succulence.

## Conclusions

Our results showed a continuum of isohydry and anisohydry, with hydroscape areas ranging from 0.72 to 7.01 MPa^2^ across the 12 woody species with leaf succulence. However, cumulative water loss before evapotranspiration ceased in drying soils was not related to degree of iso-anisohydry or leaf succulence. Turgor loss points of all 12 species were very high (−1.32 to −0.59 MPa), and there were no relationships between Ψ_TLP_ and either Ψ_pd_ when transpiration ceased or in hydroscape area. The three CAM plants, *C. modestus*, *C. rossii* and *D. crassifolium* were more isohydric with greater leaf succulence and lower root allocation. These CAM plants with thickened leaves and/or stems had smaller hydroscape areas, ceased transpiration at less negative Ψ_pd_, shortly after Ψ_TLP_, and used the stored water to maintain high water status. In contrast, the nine other woody plants ceased transpiration at more negative Ψ_pd_ and had larger hydroscape areas compared with the three CAM plants. Besides, there was a significant relationship between hydroscape area and leaf succulence among all 12 species, but when the three CAM plants were removed, the relationship was no longer significant. This suggests that greater leaf succulence can be related to isohydry, but this may have been influenced by the fact that these species with the greatest leaf succulence were also CAM plants.

## Supplementary Material

Supplementary_Tables_tpad066

## Data Availability

The authors confirm that the data supporting the findings of this study are available within the article [and/or] its supplementary materials.
