# Supplementary material for: How does leaf succulence relate to plant drought resistance in woody shrubs?
Source: Tree Physiol. 2023 May 19;43(9):1501–13. doi: 10.1093/treephys/tpad066 (PMC10652328; doi:10.1093/treephys/tpad066)
Supplement: Supplementary_Tables_tpad066 [file supplementary_tables_tpad066.docx]

**Supplementary Table 1.** Values of cumulative water loss until evapotranspiration ceased (cumulative ET at E_0_; kg), cumulative water loss until evapotranspiration ceased per shoot dry mass (cumulative ET at E_0_/Shoot dry mass; kg H_2_O kg^-1^ shoot dry mass), cumulative water loss until evapotranspiration ceased per leaf area (cumulative ET at E_0_/Leaf area; kg H_2_O m^-2^ leaf area), cumulative VPD until evapotranspiration ceased (cumulative VPD at E_0_; KPa h), water status (Ψ_pd_ at E_0_, Ψ_TLP_, and Δψ_pd_ between E_0_ and Ψ_TLP_; MPa), hydroscape area (MPa^2^), leaf succulence measures (leaf succulence quotient (g H_2_O cm^2^ leaf area g^-1^ organic matter cm^-2^ leaf area), degree of leaf succulence (g H_2_O cm^-2^ leaf area), and leaf thickness (mm)), leaf mass per area (LMA; g leaf dry mass m^-2^ leaf area), root mass fraction (RMF; g root dry mass g^-1^ plant dry mass), degree of sclerophylly corrected by ash (g leaf organic matter cm^-2^ leaf area) and morphological trait combinations (PC1, PC2) for 12 species. Species codes are in Table 1.

| **Species codes** | ***Ac*** | ***An*** | ***Ap*** | ***As*** | ***Cm*** | ***Cr*** | ***Dc*** | ***Et*** | ***Mo*** | ***Rc*** | ***Rs*** | ***Ti*** |
| --- | --- | --- | --- | --- | --- | --- | --- | --- | --- | --- | --- | --- |
| Cumulative ET at E_0_  (kg) | 1.999 | 1.980 | 2.561 | 2.009 | 2.055 | 2.591 | 1.938 | 1.811 | 2.063 | 2.666 | 2.529 | 2.459 |
| Cumulative ET at E_0_/Shoot dry mass  (kg H_2_O kg^-1^ shoot dry mass) | 21.46 | 19.91 | 26.42 | 42.33 | 27.31 | 23.38 | 30.78 | 30.84 | 34.81 | 35.88 | 30.78 | 31.04 |
| Cumulative ET at E_0_/Leaf area  (kg H_2_O m^-2^ leaf area) | 52.82 | 38.72 | 79.62 | 66.81 | 139.05 | 128.93 | 132.26 | 107.40 | 30.99 | 98.15 | 55.57 | 80.17 |
| Cumulative VPD at E_0_  (KPa h) | 123.74 | 117.49 | 129.84 | 139.34 | 107.15 | 106.16 | 102.64 | 125.57 | 117.6 | 111.36 | 102.81 | 119.87 |
| Ψ_pd_ at E_0_  (MPa) | -2.66 | -3.58 | -2.57 | -1.86 | -1.14 | -1.24 | -1.00 | -2.69 | -2.90 | -3.63 | -4.06 | -3.15 |
| Ψ_TLP_  (MPa) | -1.32 | -0.73 | -0.64 | -0.63 | -0.76 | -0.79 | -0.78 | -0.79 | -1.09 | -1.16 | -0.59 | -0.78 |
| ΔΨ_pd_ between E_0_ and TLP  (MPa) | -1.34 | -2.85 | -1.93 | -1.24 | -0.38 | -0.45 | -0.22 | -1.90 | -1.80 | -2.46 | -3.47 | -2.37 |
| Hydroscape area  (MPa^2^) | 3.19 | 3.68 | 3.38 | 2.60 | 0.72 | 1.10 | 0.83 | 2.45 | 3.07 | 4.83 | 7.01 | 3.40 |
| Leaf succulence quotient  (g H_2_O cm^2^ leaf area g^-1^ organic matter cm^-2^ leaf area) | 2.94 | 2.43 | 3.65 | 3.25 | 12.47 | 12.14 | 14.7 | 4.17 | 4.52 | 5.14 | 3.42 | 7.49 |
| Degree of leaf succulence  (g H_2_O cm^-2^ leaf area) | 0.17 | 0.16 | 0.22 | 0.21 | 3.69 | 3.37 | 2.51 | 0.54 | 0.17 | 0.31 | 0.24 | 0.60 |
| Degree of Sclerophylly corrected by ash  (g leaf organic matter cm^-2^ leaf area) | 0.06 | 0.06 | 0.06 | 0.07 | 0.30 | 0.28 | 0.17 | 0.13 | 0.04 | 0.06 | 0.07 | 0.08 |
| Leaf thickness  (mm) | 0.33 | 0.42 | 0.35 | 0.30 | 5.57 | 5.05 | 2.42 | 0.88 | 1.12 | 0.49 | 0.52 | 0.64 |
| LMA  (g leaf dry mass m^-2^ leaf area) | 12.50 | 8.21 | 8.74 | 5.16 | 31.27 | 23.59 | 16.4 | 11.08 | 5.04 | 10.9 | 7.60 | 10.23 |
| RMF  (g root dry mass g^-1^ plant dry mass) | 0.71 | 0.58 | 0.72 | 0.70 | 0.30 | 0.21 | 0.38 | 0.67 | 0.49 | 0.76 | 0.67 | 0.72 |
| PC1 | 1.35 | 1.45 | 1.50 | 1.41 | -4.72 | -4.15 | -3.18 | 0.77 | 0.86 | 1.68 | 2.07 | 0.95 |
| PC2 | -1.00 | -0.25 | -0.03 | -2.04 | -0.07 | 1.05 | -0.50 | -1.46 | -0.46 | 1.69 | 2.53 | 0.53 |

**Supplementary Table 2**. Pearson correlation among water use (cumulative ET at E_0_, ΔET between E_0_ and TLP), water status (Ψ_pd_ at E_0_, Ψ_TLP_, and ΔΨ_pd_ between E_0_ and TLP), hydroscape area, leaf succulence measures (leaf succulence quotient, degree of leaf succulence, and leaf thickness), LMA, RMF and trait combinations (PC1, PC2) for all 12 species. P values are above the blank area (right half rectangle area) and R values are below the blank area (left half rectangle area).

P

| R | Cumulative ET at E_0_ | Ψ_pd_ at E_0_ | Ψ_TLP_ | Hydroscape area | Leaf succulence quotient | Degree of leaf succulence | Leaf thickness | LMA | RMF | ΔΨ_pd_ between E_0_ and TLP | ΔET between E_0_ and TLP | PC1 | PC2 |
| --- | --- | --- | --- | --- | --- | --- | --- | --- | --- | --- | --- | --- | --- |
| Cumulative ET at E_0_ |  | 0.348 | 0.813 | 0.150 | 0.963 | 0.989 | 0.954 | 0.980 | 0.717 | 0.316 | 0.004 | 0.749 | 0.001 |
| Ψ_pd_ at E_0_ | -0.298 |  | 0.663 | <0.001 | 0.003 | 0.002 | 0.006 | 0.015 | 0.009 | <0.001 | 0.210 | <0.001 | 0.175 |
| Ψ_TLP_ | 0.076 | 0.141 |  | 0.979 | 0.699 | 0.581 | 0.690 | 0.937 | 0.702 | 0.795 | 0.881 | 0.691 | 0.859 |
| Hydroscape area | 0.442 | -0.919 | -0.008 |  | 0.011 | 0.008 | 0.015 | 0.024 | 0.013 | <0.001 | 0.276 | 0.002 | 0.062 |
| Leaf succulence quotient | 0.015 | 0.770 | 0.125 | -0.701 |  | <0.001 | 0.001 | 0.002 | 0.002 | 0.005 | 0.430 | <0.001 | 0.707 |
| Degree of leaf succulence | -0.005 | 0.800 | 0.177 | -0.724 | 0.920 |  | <0.001 | <0.001 | 0.001 | 0.011 | 0.855 | <0.001 | 0.750 |
| Leaf thickness | 0.019 | 0.736 | 0.129 | -0.682 | 0.827 | 0.921 |  | <0.001 | <0.001 | 0.009 | 0.813 | <0.001 | 0.700 |
| LMA | 0.008 | 0.680 | 0.026 | -0.642 | 0.792 | 0.935 | 0.931 |  | 0.004 | 0.015 | 0.983 | <0.001 | 0.701 |
| RMF | 0.117 | -0.718 | -0.124 | 0.687 | -0.803 | -0.817 | -0.911 | -0.755 |  | 0.012 | 0.408 | <0.001 | 0.861 |
| ΔΨ_pd_ between E_0_ and TLP | -0.317 | 0.975 | -0.084 | -0.923 | 0.747 | 0.702 | 0.712 | 0.679 | -0.694 |  | 0.193 | <0.001 | 0.157 |
| ΔET between E_0_ and TLP | 0.763 | -0.390 | 0.049 | 0.342 | -0.252 | -0.059 | -0.077 | -0.007 | 0.264 | -0.404 |  | 0.465 | 0.100 |
| PC1 | 0.103 | -0.847 | -0.128 | 0.797 | -0.927 | -0.989 | -0.959 | -0.907 | 0.92 | -0.824 | 0.233 |  | 1.000 |
| PC2 | 0.810 | -0.420 | 0.057 | 0.554 | 0.121 | 0.103 | 0.124 | 0.124 | -0.057 | -0.435 | 0.498 | <0.001 |  |
